# Advancing Healthcare Sustainability through Decision Support

**DOI:** 10.1055/s-0046-1816619

**Published:** 2026-11-03

**Authors:** Titus Schleyer, Genevieve S. Silva, Jyothi Tirumalasetty, Shivam Vedak, Haseena Rajeevan, Muhammad Muinul Islam, Beenish Moalla Chaudhry, Manijeh Berenji

**Affiliations:** 1 Regenstrief Institute, IndianapolisUSA; 2 Indiana University School of Medicine, IndianapolisUSA; 3 University of California San Francisco (UCSF) Health, San Francisco, CaliforniaUSA; 4 Stanford University School of Medicine, Stanford, CAUSA; 5 Stanford University School of Medicine, Stanford, CAUSA; 6 Yale University School of Medicine, New Haven, CTUSA; 7 University of Missouri, Columbia School of Medicine, MOUSA; 8 University of Louisiana at Lafayette, School of Computing and Informatics, Lafayette, LAUSA; 9 University of California Irvine School of Medicine Irvine CAUSA; 10 University of California Irvine Joe C Wen School of Population and Public Health Irvine CAUSA; 11 University of California Center for Climate Health and Equity San Francisco CAUSA

**Keywords:** Sustainability, Decision Support Systems, Healthcare Delivery, Climate Change, Informatics, Environment

## Abstract

**Objectives** : Healthcare is a resource-intensive industry that contributes to pollution and environmental harm. We are in the early stages of understanding how decision support in healthcare can help address these challenges. This paper examines the role that decision support, at all levels of healthcare, could play in achieving more sustainable healthcare.

**Methods** : We synthesized the body of literature on healthcare, decision support, and environmental harm/climate change through two Medline searches. The searches focused on healthcare, decision support, informatics, climate change, sustainability, and related terms.

**Results** : The literature search yielded 238 papers related to climate change and informatics, but only six of those described clinical decision support systems. In order to describe the opportunities for decision support beyond the narrow corpus of six papers, we used the six priority sustainability areas identified by the Agency for Healthcare Research and Quality (AHRQ): energy, transportation, pharmaceuticals and chemicals, anesthetic gases, medical devices and supplies, and food.

**Conclusions** : While there are significant opportunities to advance sustainability in healthcare, developed and implemented decision support is available for only a small fraction of them. Therefore, the informatics community worldwide should craft a comprehensive and systematic agenda for developing, implementing, and evaluating decision support tools and algorithms for sustainability. This agenda should address all emission scopes; be tailored to different stakeholders; relate sustainability to quality of care; include the development of centralized and accessible knowledge bases; work in concert with other ways to advance awareness of and engagement, including building a culture of sustainability; and encompass creating a worldwide informatics community to advance healthcare sustainability.

## 1. Introduction

Healthcare is a resource-intensive industry that contributes to pollution and environmental harm [ 1 ], with significant effects on human [ 2 ] and planetary health [ 3 ]. Healthcare workers are concerned about their industry's effect on the environment and the climate, and expect their employers to address healthcare's contribution to climate change [ 4 ]. Most, if not all, decisions in healthcare, such as construction and operation of facilities, heating and cooling of buildings, sourcing and use of supplies and equipment, procurement of food, diagnosing and treating patients, and management of waste, have sustainability implications. Informatics is only in the early stages of understanding how decision support [ 5 6 ] can help healthcare advance sustainability and reduce environmental harm. This paper examines what role decision support, at all levels of healthcare (e.g. patient/clinician, unit, facility, service and health system), could play in rendering healthcare sustainable.

The World Health Organization defines an environmentally sustainable health system as *“a health system that improves, maintains or restores health, while minimizing negative impacts on the environment and leveraging opportunities to restore and improve it, to the benefit of the health and well-being of current and future generations” [ 7 ]. Mortimer et al. [ 8 ] suggest that sustainability be “considered a domain of quality in healthcare, extending the responsibility of health services to patients not just of today but of the future.”* This view of healthcare being intrinsically linked to sustainability is echoed by other authors [ 9 10 ].

Decision support is typically understood by informaticians to mean “clinical decision support” (CDS), with a long history of development, implementation, and evaluation [ 5 6 ]. Work on CDS specifically related to sustainability is relatively scarce. Therefore, in this paper we apply the more general lens of *decision support* as a term for processes, tools, and systems designed to assist individuals or organizations in making informed, data-driven choices [ 11 ].

As of 2020, healthcare contributed 8.5% of greenhouse gas (GHG) emissions in the United States (US) and 4.5% globally [ 12 ]. Environmental pollution and climate change negatively affect personal, public, and ecosystem health [ 2 3 13 ] - yet, the healthcare sector continues to compromise its core mission to make and keep people healthy by contributing to pollution. In response, multiple efforts to advance sustainability in healthcare have been launched. In the US, these include the Climate and Health Program [ 14 ] of the Centers for Disease Control and Prevention, the TEAM Decarbonization and Resilience Initiative [ 15 ] of the Centers for Medicare and Medicaid Services, the Center for Climate, Health, and Equity [ 16 ] of the American Public Health Association, the National Academy of Medicine's Action Collaborative [ 17 ] on Decarbonizing the US Health Sector, the Medical Society Consortium on Climate and Health [ 18 ], and Health Care Without Harm [ 19 ]. Global programs include the Planetary Health Alliance [ 20 ], the World Health Organization Alliance for Transformative Action on Climate and Health [ 21 ], and the Global Consortium on Climate and Health Education [ 22 ].

Sources of pollutants in healthcare include healthcare emissions, which are categorized as Scope 1, 2, and 3 [ 23 ] (see Table 1 ), depending on where the emissions originate. Scope 1 includes *direct emissions* , Scope 2 *indirect energy emissions* , and Scope 3 *indirect emissions from the value chain* . Figure 1 shows an overview of healthcare GHG emissions.

**Table 1.** Definitions of greenhouse gas emission scopes (from The Greenhouse Gas Protocol, a Corporate Accounting and Reporting Standard [ 23 ] HC = Healthcare).

Scope	Description	Examples	% of US HC emissions
1	Emissions from sources directly owned or controlled by the healthcare organization	Combustion of fossil fuels for heating, cooling, and power generation Emissions from hospital-owned vehicles On-site waste incineration	10
2	Emissions resulting from the generation of purchased electricity, heating, and cooling consumed by the organization	Electricity use for Lighting, heating and HVAC systems Medical equipment Decision support systems, especially if based on advanced machine learning algorithms, and required IT infrastructure Purchased steam or chilled water for facility operations	8
3	Emissions that are a consequence of the organization's activities but occur from sources it does not own or directly control	Manufacturing and transportation of medical supplies, pharmaceuticals, and equipment Patient and staff commuting Waste disposal and treatment Investments and outsourced services (e.g., laundry, food services)	82

**Figure 1.**
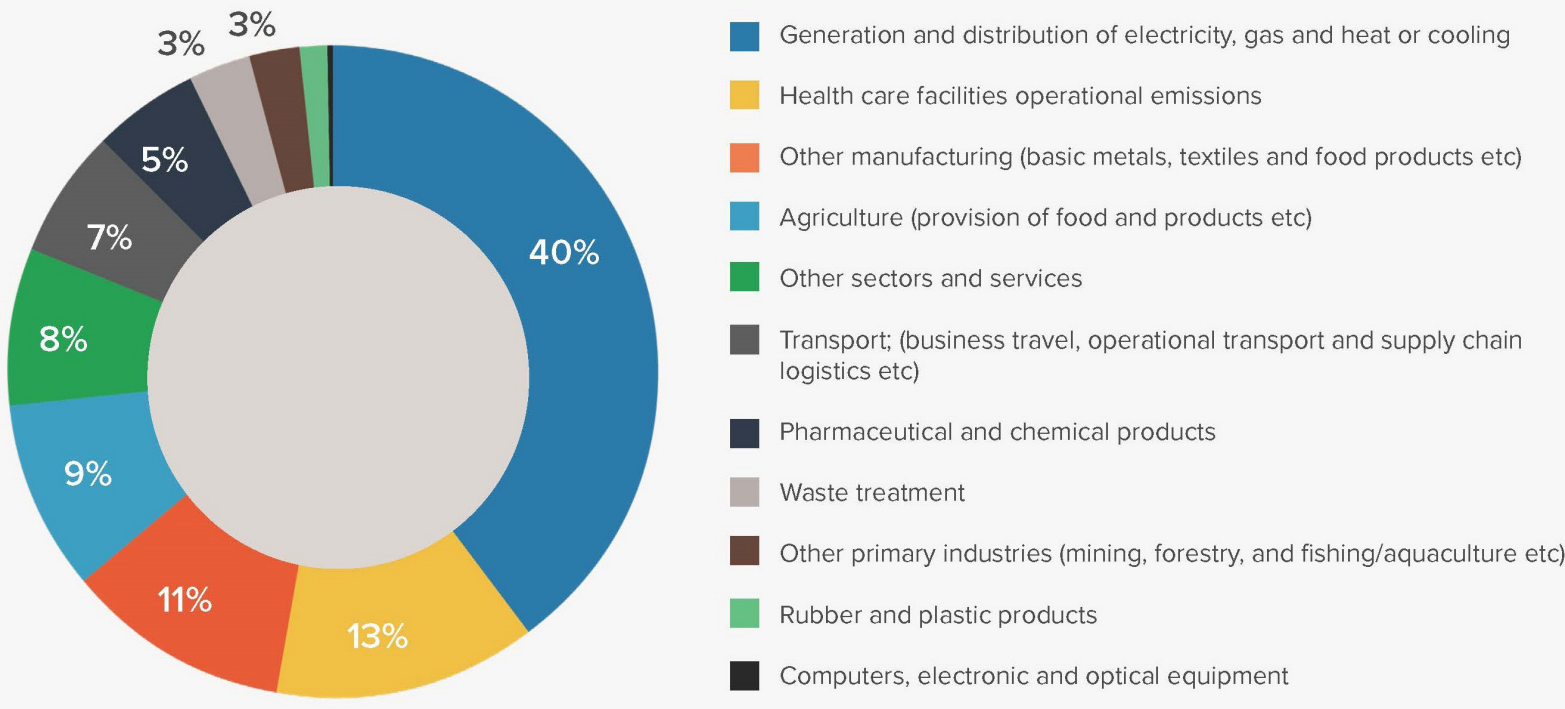
Global health care GHG emission split by production sector [ 24 ]. © Health Care Without Harm 2025. Used with permission.

To date, the question of how and to what degree decision support tools of any kind could help healthcare work towards sustainability remains unanswered. Decisions in healthcare that affect sustainability are made at multiple levels, such as the patient/clinician, hospital unit, facility, service, and health system levels, and are influenced by regulatory, professional, cultural, financial, ethical, and other factors. Decision-making tools, specifically for clinical decision support (CDS), have historically and currently been the domain of biomedical informatics [ 5 6 25 ].

Beyond decisions made in the context of patient care, most decisions made in healthcare have environmental effects. Therefore, it makes sense to examine healthcare decision-making support through a broader lens, ranging from the individual (clinical) to the systems level. This review surveys the literature on healthcare sustainability that could inform the design, development, and implementation of decision support tools, critically evaluate contributions made by decision support to enhance sustainability, and articulate how decision support for sustainability should evolve.

## 2. Methods

To review the literature on decision support systems and healthcare sustainability, we performed a literature search and article selection following the PRISMA-ScR guidelines [ 26 ]. To retrieve relevant literature, we first queried PubMed for the Medical Subject Headings (MeSH) terms [ 27 ] “climate change” and “informatics” in July 2024. Two authors (TS and MMI) reviewed all citations independently for inclusion and resolved conflicts jointly. The search and its results are briefly described in Schleyer *et al.* [ 28 ]. Upon reviewing the MeSH terms for several articles relevant to our focus, it became apparent that “climate change” was not consistently assigned as a keyword, resulting in relevant citations being missed in our initial search. Consequently, we conducted an expanded search of MEDLINE, using a combination of MeSH terms and keywords to retrieve papers related to healthcare, decision support, informatics, climate change, sustainability, and related terms. Author HR screened the resulting papers and authors HR and BC performed the full-text review of initially relevant papers from both searches. *Appendix 1* documents the detailed searches as executed.

## 3. Results

The search for “climate change” and “informatics” yielded 1,823 citations, dating back to 1989. Of those, 126 focused on climate, health, and informatics [ 29 ]. The expanded search yielded 9,515 citations (after removing 20 duplicates from the first search review results). Of these, 9,403 were excluded, leaving 112 citations that addressed healthcare sustainability and informatics. The 126 and 112 citations from the initial and enhanced searches, respectively, were combined into one result set for full-text review. Review of this set yielded six papers that described the implementation of a decision support intervention for sustainability. Figure 2 shows the PRISMA diagram for the search and review, and Table 2 presents a summary of the six papers.

**Figure 2.**
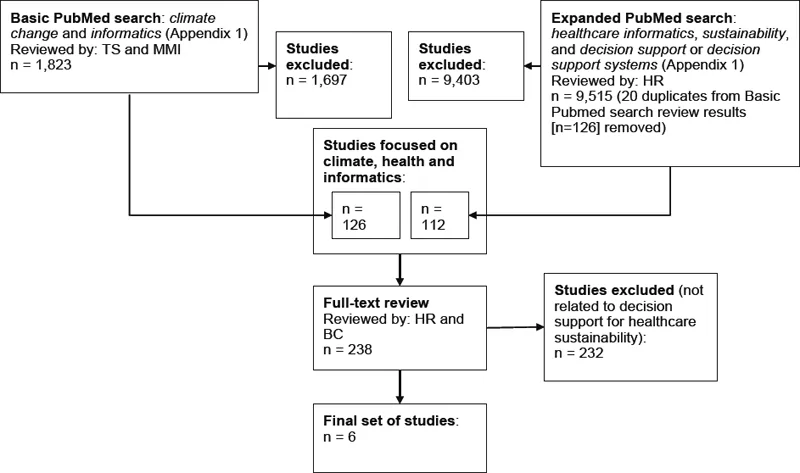
PRISMA flowchart of the search and review process.

**Table 2.** Clinical decision support implementations for sustainability.

Study	Implemented in	Evaluation period	Intervention	Outcome
Olmos 2024 et al. [ 96 ]	Epic Anesthesia Information Management System	08/2018 to 12/2019	Alerts providers when fresh gas flows (FGFs) exceeded recommended levels during anesthesia maintenance	Significant reduction in mean FGF. Sevoflurane= decreased by 0.6 L/min Desflurane= decreased by 0.2 L/min
Woolen 2023 et al. [ 39 ]	Power management strategies for MRI scanners	time period: 39 days	Switches MRI units from idle to off mode for 12 hours overnight	Cut power consumption by 25%–33%, saving 12.3–21.0 MWh annually, $1,717–$2,943, and 8.7–14.9 metric tons of CO _2_ eq
Collins 2024 et al. [ 88 ]	Within the electronic health record system, USA	07/2023 to 07/2024	Alerts providers when the 10-minute average FGF exceeded 1 L/min	Achieved a 36.7% reduction in sevoflurane usage
Schranz 2024 et al. [ 48 ]	Point-of-care (POC) Clinical Decision Support Tool (CDST) that incorporates guidance directly into the physician workflow	2019 and 2021	Examines the relationship between imaging referrals pre- and post-POC CDST implementation	Reduction in absolute advanced imaging volume = 8.2% duplicate CT and MRI imaging=62% reduction in carbon emissions=13.5%
Johnson 2024 et al. [ 99 ]	Propofol volume and dosing decision support tool implemented in Epic	01/2021 to 06/2021 and 01/2023 to 10/2023 for two different procedures	Estimates the volume of propofol saved using the decision support tool	Estimation that with 25% adherence to the tool, 500mL of propofol waste could have been saved.
Ramaswamy 2022 et al. [ 97 ]	FGF CDS system successfully deployed at five UC Health campuses	08/2018 to 05/2022	Shows that the CDS system can be implemented in other medical facilities using the toolkit	FGF CDS system implemented across five UC Health campuses

Of those six papers ( Table 2 ), four focused on anesthesiology, addressing the tracking of fresh gas flow to minimize anesthetic waste and the reduction of propofol waste. (Two papers related to the same project.) The remaining two papers, from radiology, discussed managing radiology referrals and operational strategies using CDS tools. The strength of the papers lay in the fact that all systems were implemented, one was scaled across several sites in a health system, all addressed practical and relevant problems in sustainability, and all except one calculated actual CO2 emission savings. The papers highlighted collaboration among clinicians, informaticians, and sustainability experts as crucial for the effective implementation and evaluation of these tools. However, the studies also had weaknesses. Five of the six were limited to single-site pilot or short-term intervention, constraining their generalizability and long-term effect. The lack of control groups and/or randomization made it challenging to attribute observed changes solely to the CDS intervention. Additionally, sustainability of user behavior changes over time and potential disengagement were not addressed. Despite mentioning cost savings, comprehensive budget effect evaluations, including implementation costs, were missing. Lastly, Johnson *et al.* 's study calculated anticipated propofol rather than actual waste reduction and lacked validation in real-world clinical settings.

To provide a broader context of how decision support could advance healthcare sustainability beyond the six papers our search identified, we review decision support opportunities for the six priority sustainability areas identified by the Agency for Healthcare Research and Quality (AHRQ): energy, transportation, pharmaceuticals and chemicals, anesthetic gases, medical devices and supplies, and food [ 30 ]. The National Academy of Medicine [ 31 ] added waste and single use plastic reduction, which we address in our discussion of supplies.

### 3.1 Energy

Minimizing energy consumption in healthcare facilities is a pivotal strategy to curtail environmental effects. Heating, ventilation, and air conditioning (HVAC) systems remain central to these efforts, as they account for nearly half of energy usage in hospitals [ 32 33 ]. Implementing technologies such as heat pumps and heat recovery ventilation, along with architectural design solutions to lessen heating and cooling demands, can significantly reduce energy expenditures and emissions. Healthcare organizations are also increasingly adopting renewable energy sources such as solar, wind, and geothermal systems to diminish their carbon footprints while enhancing energy resilience. The UK National Health Service (NHS) has committed to achieving net-zero carbon emissions by 2040, in part through expanding on-site renewable energy generation and upgrading facility infrastructure. US health networks like Kaiser Permanente have similarly pledged to reduce emissions by integrating solar arrays and improving building energy efficiency [ 34 35 36 ]. While these examples illustrate the feasibility and potential of large-scale renewable adoption and building energy optimization, widespread adoption remains inconsistent.

Within healthcare organizations, medical imaging devices such as MRI and CT scanners contribute significantly to energy consumption [ 37 ]. A substantial portion of the energy used—ranging from 40% to 91%—is estimated to be non-productive, occurring when devices are powered on but not actively in use [ 38 ]. Organization-level interventions (e.g., employing an MRI device power-save mode overnight in outpatient units) have been estimated to save up to $10 million and 54,000 MTCO2eq nationally [ 39 ]. The recently-published *Sustainability Criteria for Purchasing Medical Imaging Equipment* [ 40 ] highlights a role for sector-level supply chain management to prioritize equipment with the capacity for automated or programmable energy-save modes.

Recent advances in artificial intelligence (AI) offer additional opportunities to optimize energy usage. Monitoring real-time data, from patient census fluctuations to local weather conditions, can allow for AI-driven recalibration of HVAC operations, leading to measurable energy savings [ 41 42 43 44 ]. For instance, hospitals leveraging AI for predictive control of operating room temperatures have realized meaningful efficiency gains without compromising patient care, stabilizing indoor air quality while lowering utility expenditures [ 45 ].

These energy-conservation innovations intersect with decision support systems at clinical and organizational levels. At the clinical level, AI tools can assist with management of operating rooms [ 45 ] by predicting surgical case durations, optimizing post-anesthesia care unit resource allocation, and detecting surgical case cancellations. In addition, the American College of Radiology (ACR) [ 46 ] and European Society of Radiology (ESR) [ 47 ] established practice guidelines for CDS systems to facilitate appropriate imaging orders. Integrating AI tools within these systems can further conserve energy by optimizing imaging utilization. Implementing a point-of-care CDS tool within a radiology department led to an 8.2% decrease in imaging volume and a 61% reduction in duplicative imaging reducing extraneous patient radiation exposure and carbon emissions [ 48 ]. At the organizational level, dashboards and analytics platforms can facilitate oversight of hospital-wide energy consumption, allowing administrators to pinpoint inefficiencies, project cost savings, and benchmark progress toward sustainability targets. Such dashboards and automated protocols for powering down equipment or using power-save mode during periods of inactivity can significantly reduce the environmental footprint of hospitals' radiology departments [ 39 49 ]. Some institutions now embed renewable energy contributions into energy management dashboards to visualize the effect of solar or wind power on overall facility usage, thereby fostering data-driven decision-making and continuous improvement [ 50 51 52 ].

Collectively, these strategies highlight the substantial gains possible from applying decision support tools to energy optimization in healthcare. Evidence demonstrates that targeted HVAC enhancements, renewable energy adoption, and data-driven management systems can curb environmental footprints while preserving or even enhancing patient care. As healthcare facilities continue to explore AI-driven solutions, aligning operational decisions with sustainability objectives becomes increasingly feasible. However, it is essential to acknowledge the current energy intensity of using AI itself [ 53 54 55 56 57 ]. The growth of AI has led multiple large technology companies to delay or abandon their decarbonization goals [ 58 ], and led to increased energy demand. These tools should be employed judiciously and with the goal of maximizing decarbonization gains within healthcare.

### 3.2 Transportation

Telehealth emerged as a notable strategy for limiting healthcare's transportation-related emissions by reducing patient and provider travel, when clinically appropriate [ 59 60 61 62 63 ]. One program reported a substantial decrease in transportation-associated carbon emissions after large-scale telehealth adoption [ 59 ], while widespread reliance on telehealth during the COVID-19 pandemic was linked to measurable declines in emissions in urban health systems [ 51 64 65 ]. These findings are supported by various studies demonstrating significant reductions in healthcare's carbon footprint with sustained telehealth implementation, especially in rural areas where long travel distances are common [ 60 61 62 63 66 67 68 ]. In addition to decreasing patient transportation emissions, telehealth can also expand healthcare access and reduce wait times. Disparities in telehealth access remain a concern, highlighting the need for equitable deployment across different populations. It is also critical to ensure that telehealth is only offered when clinically appropriate, prioritizing clinical equipoise and patient outcomes.

Integrated CDS offers a potential solution to the above challenges in telehealth implementation, as it can identify patients who would most benefit from virtual appointments based on clinical criteria, geography, and resource availability. One health system, for example, employed predictive analytics to prioritize virtual visits for patients with chronic conditions, reducing repeated in-person consultations and curtailing overall vehicular travel [ 69 70 ]. Further, CDS could be leveraged to prioritize immunocompromised patients for telehealth. At the organizational level, integrating telehealth usage metrics into decision support dashboards can enable administrators to visualize environmental benefits. Kaiser Permanente reported that tracking fuel consumption and carbon savings alongside clinical metrics helps align institutional goals with broader sustainability objectives [ 36 71 ]. Consequently, telehealth may represent a feasible and effective approach to decrease healthcare's environmental footprint by minimizing unnecessary trips and optimizing resource allocation [ 72 73 74 ].

### 3.3 Pharmaceuticals and Chemicals

The pharmaceutical industry's environmental effect extends throughout the value chain, from the creation of a medication to its distribution to the pharmacy/medical establishment and, ultimately, the end user. Environmental concerns arise from the resources and energy needed to manufacture the product and the potentially harmful chemical byproducts requiring disposal [ 75 ]. Additionally, the industry faces challenges in packaging and transportation, particularly for temperature-sensitive products like biologicals which typically require temperature-controlled polystyrene packaging and distribution mechanisms (also known as cold-chain) [ 76 ].

Green supply chain initiatives have reduced environmental effects across production, distribution, and disposal stages [ 77 ]. Companies such as Pfizer and Johnson & Johnson adopted strategies such as renewable energy utilization, waste reduction, and eco-friendly packaging, contributing to measurable improvements in sustainability metrics [ 78 79 ]. A partnership between Boston Medical Center and Takeda focuses on monitoring and reducing drug packaging waste [ 80 ]. Other organizations, such as the Circularity in Primary Pharmaceutical Packaging Accelerator, are reducing pharmaceutical packaging waste and delivering aspects of circularity, focusing on blister packs and injector pens [ 81 ]. While specific reductions vary by context, green chemistry principles and solvent recovery processes offer additional pathways for further emissions mitigation [ 77 ].

For the pharmacy/medical establishment and end users, the disposal of expired medications and regulatory requirements for discarding partially used products contributes to environmental effect, which highlights opportunities to improve inventory management systems and reordering practices [ 82 ]. Pharmaceutical products themselves may also have a substantial footprint [ 83 ]. Pressurized metered-dose inhalers (pMDIs) are responsible for approximately 0.03% of yearly global GHG emissions, since one pMDI (200 doses) has a carbon footprint equivalent to a 180-mile (290-km) automobile ride [ 84 ]. The hydrofluorocarbon propellants used to aerosolize pMDIs have very high global warming potential (GWP), with a carbon footprint 30 times higher than dry-powder and soft-mist inhalers. Despite widespread availability of lower-emissions alternatives such as dry-powder and soft-mist inhaler devices, pMDIs were responsible for nearly 70% of inhaler prescriptions in the US and 98% of inhaler-related carbon emissions in 2022. European and Canadian health systems have implemented widespread initiatives to educate clinicians and promote lower-emissions inhaler prescribing, while other countries like the US lag behind on such programs [ 85 ].

Despite progress, the comprehensive adoption of pharmaceutical green supply chain strategies remains limited. Existing data often focus on specific interventions rather than holistic transformations, and longitudinal studies quantifying the cumulative effects of these measures are still emerging. Digital tools, including CDS systems, could play a pivotal role by embedding environmental criteria into prescribing workflows guiding clinicians toward safer alternatives, and minimizing active pharmaceutical ingredient (API) contamination in water bodies. The carbon footprint of inhaler devices in the US, along with asthma guidelines, cost data, and insurance formulary data could be leveraged to deliver non-interruptive alerts in the electronic health record to encourage clinicians to consider lower-emissions inhalers when clinically appropriate [ 86 87 ]. CDS tools have been successfully implemented in initiatives to reduce the carbon footprint of anesthesia [ 88 ] and will be a pivotal next step in implementing inhaler decarbonization programs [ 89 ]. At an organizational level, integrated decision support dashboards tracking pharmaceutical inventory, emissions, and waste enable data-driven strategies to streamline procurement, optimize production, and enhance waste management practices.

### 3.4 Anesthetic gases

The contribution of anesthetic gases to healthcare's carbon footprint is relatively small (1% of all US healthcare GHG emissions in 2012) [ 90 ], but is exacerbated by the fact that anesthetic gases include potent GHGs: Desflurane's GWP is 2,540 times greater than carbon dioxide's, exceeding that of other inhaled anesthetics such as sevoflurane and isoflurane [ 91 ]. Strategies such as low-flow anesthesia, adopting total intravenous anesthesia (TIVA), and switching to lower-GWP agents like sevoflurane can substantially reduce emissions [ 92 ]. Educating anesthesiologists about environmentally-preferred anesthetic choices and avoiding excessive fresh gas flow rates have been shown to reduce ozone depletion potential (ODP) and GHG emissions by 65–95% for abdominal and vaginal hysterectomies [ 93 ]. Switching to propofol or other intravenous (IV) or regional anesthesia techniques, when clinically appropriate, can reduce ODP by 3% in laparoscopic and 28% in robotic hysterectomies [ 93 ]. However, systemic barriers, including behavioral inertia and inconsistent policy implementation, limit the widespread adoption of these practices [ 94 ]. Low-flow anesthesia [ 95 96 97 ] techniques effectively reduce gas consumption, with studies suggesting substantial reductions without compromising patient safety [ 91 ], while advanced scavenging systems can capture and neutralize waste gases. Similarly large reductions in GHG emissions are attainable for nitrous oxide, for which delivery through built-in pipe systems results in the loss of nearly 90% of the gas before it reaches the patient [ 98 ]. Although robust data affirm the effectiveness of these mitigation strategies, barriers to sustainable anesthesia include behavioral inertia among clinicians, systemic gaps in policy, and inconsistent implementation of mitigation technologies [ 94 ].

CDS systems could foster sustainable practices by alerting anesthesiologists to agents with high GWP and suggesting viable alternatives [ 91 96 99 ]. Such tools could follow an algorithmic approach based upon provider sustainability checklists published by the American Society of Anesthesiologists and function similarly to “care pathways” designed for common patient management scenarios at large health systems [ 100 ]. This type of evidence-based guidance could help clinicians to tailor anesthesia to individual patient and surgical needs while mitigating environmental impact. At the organizational level, decision support platforms that track anesthetic gas usage and emissions provide hospital administrators with crucial data to benchmark progress against sustainability objectives, such as phasing down use of nitrous oxide and desflurane.

### 3.5 Medical Devices and Supplies

Another critical component of healthcare procurement involves medical devices and supplies that contribute significantly to healthcare's supply chain-associated environmental footprint. This effect is attributable in part to manufacturing and transport but also due to disposal and end-of-life processing. Single-use disposable items made from materials such as polypropylene and other plastics require substantial energy for production and disposal [ 93 ]. Lower-effect alternatives, including reusable surgical instruments and supplies and reprocessed single-use devices, demonstrate potential for mitigating these effects without exposing patients to increased risk of infection [ 101 102 ]. Life cycle assessments comparing reusable to single-use materials for an array of products, including isolation gowns, speculums, laryngoscopes, and pulse oximeters have consistently found that reusables lead to lower GHG emissions [ 103 104 105 106 107 108 ], even after accounting for disinfection and processing. Studies that reported water and waste reductions had savings from 41-57% and 93-97%, respectively [ 103 104 105 106 107 108 ]. Another source of environmental effect comes from wasteful procedural setup, in which standardized sets of sterile items are opened and not or only partially used, still requiring disposal [ 109 ]. Interventions to streamline surgical setup and biopsy kits have achieved a 22% reduction in GHG emissions per surgical procedure and an 84% reduction in kits with wasted supplies [ 93 110 ]. Further gains are achievable through integrated waste management strategies and improved stewardship of regulated medical waste, which is frequently overused and requires greater emissions and costs to process [ 93 111 112 113 ] than regular waste. Finally, institutional investments in alternative materials have led to reduced environmental effects. One study highlighted a 30% decrease in waste in a large urban hospital following the adoption of biodegradable gloves and syringes, aligning with a 42% potential reduction in GHG emissions when combined with other sustainability interventions [ 110 113 114 ].

CDS systems may further facilitate sustainable practices by flagging disposable items and recommending eco-friendly alternatives. CDS platforms integrated into Singapore's National University Health System procurement systems reduced single-use material reliance by 25% through a focus on reusable and recyclable items [ 115 116 ]. Such platforms can help staff to calibrate required procedural setup to align with patient arrival time and anticipated procedure complexity to avoid opened but unused supplies. At the organizational level, decision support dashboards that track procurement decisions and quantify associated environmental effects have shown significant promise. The NHS's centralized monitoring system achieved an 11% reduction in healthcare-related GHG emissions over five years, leveraging tools such as material flow analysis and life cycle assessment (LCA) metrics to identify inefficiencies and optimize resource use [ 110 117 ]. Department-level purchase tracking systems may also be used to quantify supplies used per procedure type and volume to optimize supply reordering systems. Hospital data management systems that track monthly volumes across distinct waste streams and departments are key for reducing system waste production [ 118 119 ]. AI-powered platforms designed for healthcare waste stream auditing demonstrated increased efficiency in waste categorization and marked decreases in volume of healthcare waste [ 120 ]. Together, these findings emphasize the potential of combining eco-friendly supply chain innovations with targeted decision support mechanisms to reduce healthcare's environmental burden effectively.

### 3.6 Food

Food in healthcare institutions is responsible for about 9% of US healthcare's carbon footprint [ 24 ]. Hospitals are increasingly adopting sustainable meal programs, emphasizing local sourcing, plant-forward menus, and food waste reduction. These efforts are supported by initiatives like the Healthy Food in Health Care initiative, which collaborates with institutions nationwide to adopt eco-friendly food procurement policies [ 121 ]. A case study at Stanford Health Care reported a substantial decrease in GHG emissions by prioritizing locally produced and seasonal ingredients, aligning with evidence that sustainable procurement can reduce emissions by up to 30% across healthcare institutions [ 121 122 123 ]. Similarly, the University of California health systems achieved 26% sustainable food procurement in 2019, which contributed to reduced GHG emissions across their institutions [ 124 ]. These cases support the broader consensus that ruminant meats, particularly beef and lamb, contribute disproportionately to healthcare-related emissions, with meat-heavy menus accounting for up to 60% of food-related emissions and beef alone emitting over 26 kg CO2-eq/kg [ 121 125 126 ]. Moreover, the industrial food system accounts for approximately 10.5% of the nation's energy use, underscoring the significant environmental effect of current practices [ 121 ]. Transitioning to plant-forward options has proven effective for lowering the carbon footprint of hospital food systems, given that grains, legumes, and vegetables can have median emissions as low as 0.37 kg CO2-eq/kg, and swapping beef with chicken or vegetarian alternatives can reduce emissions by 8- to 20-fold [ 125 126 ]. This shift aligns with California's “Cool Food Pledge,” which seeks to reduce the climate effect of food procurement by prioritizing low-emission dietary choices [ 124 ]. Such initiatives also represent dual wins for improving environmental sustainability and patient health. For instance, Boston Medical Center operates two rooftop farms from which physicians may “prescribe” produce to supplement diets and support the health of patients with chronic disease. These farms also prevent stormwater runoff and reduce energy use [ 127 ].

Healthcare organizations are also tackling food waste through composting, donation, and other diversion strategies. Some institutions have implemented comprehensive tracking systems and redistributing of surplus food to community programs [ 122 128 ]. Other healthcare systems have implemented a room service meal model for patients to reduce waste of unwanted food items, leading to a decrease in plate waste from 33% to 9% at one system [ 129 ]. Over 250 hospitals have signed the Health Care Without Harm pledge to minimize food waste, reflecting a nationwide commitment to sustainable practices [ 121 ].

Nonetheless, implementation challenges remain, including stigma around plant-based diets, a lack of consistent policy frameworks to encourage adoption, and infrastructure challenges around changing existing hospital operations and staff workflow [ 122 128 ]. CDS systems can bridge these gaps by integrating sustainability data with patient nutritional requirements and facilitating plant-forward meal planning when clinically appropriate. At an organizational level, decision support tools may be implemented to reduce the volume of wasted, uneaten food by designing inpatient meal selection systems to optimize “opt-in” rather than “opt-out” ordering (within clinically-determined guidelines for those on special diets). These platforms could be modeled after the online tools developed by organizations implementing medically-tailored meals for patients [ 130 ]. Decision support dashboards that track sourcing decisions and measure food-related emissions could also enable administrators to establish sustainability benchmarks and guide strategic interventions. Collectively, these highlight the substantive contribution of food-related initiatives to achieving healthcare sustainability goals.

## 4. Discussion and Recommendations

This review's goal was to describe contributions made by decision support to enhance sustainability, survey the literature on healthcare sustainability that could inform the design, development, and implementation of decision support tools, and articulate how decision support for sustainability should evolve. With six papers focused on two narrow domains (see Table 2 ), i. e. anesthesia and radiology, the current literature on decision support for sustainability in healthcare is negligible. Yet, as our review of the AHRQ's six priority sustainability areas [ 30 ] showed, there are significant opportunities for decision support to help advance healthcare sustainability. The Inset presents seven recommendations for informatics, several of which are aligned with recommendations for general environmental decision support systems [ 131 ]. We discuss the recommendations briefly below.

First, informatics should develop a comprehensive and systematic agenda for advancing decision support for sustainability (Recommendation 1). This requires a broader read of “decision support” than informatics typically understands it (as in “clinical decision support”). Many, if not all decisions, for how to design and operate healthcare have sustainability implications. Those decisions range from how we construct and operate our facilities, use our resources such as clinics and equipment, configure patient schedules, manage waste, and feed our patients and visitors to how we diagnose and treat disease. This requires that decision support should reduce emissions across Scopes 1, 2 and 3 (Recommendation 2). In addition, sustainability decisions are made across all areas of health system operations by many individuals, groups, and entities. Tailoring decision support tools to the specific needs of these stakeholders, for instance through point-of-care alerts and advice, clinical dashboards, predictive analytics, system-wide metrics, and organizational procurement tools, can ensure that sustainability is considered across all levels of decision-making (Recommendation 3).

**Table:** 

Inset: Recommendations for informatics to advance decision support for healthcare sustainability
• Craft a comprehensive and systematic agenda for developing, implementing, and evaluating decision support tools and algorithms for sustainability. • Develop decision support tools to reduce emissions across Scopes 1, 2, and 3. These decision support tools should consider any and all data, ranging from facility construction, efficiency, and operations to diagnosis and treatment, in their analyses. For Scope 3, the lion's share of emissions, tools should enable data-driven decision making across supply chains, procurement, transportation, waste management, and outsourced services. • Take the perspectives of different stakeholders across healthcare organizations into account when developing decision support systems. Tailoring decision support tools to the specific needs of these stakeholders can ensure that sustainability is considered across all levels of decision-making. • Develop models for assessing the effect of sustainability on quality of care, with particular emphasis on approaches that have the potential to improve quality of care. • Focus on provisioning centralized and accessible knowledge bases that provide information relevant to sustainability. These knowledge bases can be integrated into decision support systems and provide healthcare organizations with data-driven insights to guide sustainable practices. • Design decision support tools in concert with other ways to advance awareness of and engagement with sustainability, such as education and policy. Help build a culture of sustainability. • Contribute to building local, regional, national, and global communities for advancing sustainability in healthcare.

Second, sustainability efforts should improve quality of care whenever possible, or at least maintain clinical equipoise (Recommendation 4). For example, improving environmental sustainability via resource conservation may reduce unnecessary procedures or unwarranted variation in care or increase care efficiency for patients, enhancing the value of care. Telemedicine, for instance, decreases patient transportation emissions while expanding healthcare access and reducing infection risks and wait times. Energy-efficient hospital designs create healthier indoor environments, and waste reduction strategies in operating rooms improve workflow, reduce costs, and optimize resource utilization. This suggests that sustainability efforts should be seen as interconnected with quality of care [ 8 ], requiring assessment of patient safety, clinical effectiveness, operational efficiency, and overall healthcare outcomes. Decision support tools should therefore incorporate data-driven evaluations of how sustainability initiatives affect patient outcomes, health co-benefits (e.g. those of fresh produce grown on a health system rooftop farm) [ 132 ], workflow efficiency, and healthcare costs.

Third, we should accelerate the development of the foundational knowledge base in healthcare sustainability that underlies effective decision support (Recommendation 5). LCAs are one source of such robust data [ 133 ] which evaluate multiple dimensions of the environmental effect of a product or service throughout its lifetime - from raw material extraction to manufacturing, distribution, use, and waste disposal. However, developing LCAs is not trivial – they require a detailed, time-consuming audit of all resources associated with a product or process. The labor-intensity of obtaining new LCA data should be balanced with their expected relative utility and novelty, considering the repository of published healthcare LCA data [ 134 ]. Where available [ 135 136 137 ], LCA data should be integrated into decision support tools. Where LCAs have not yet been conducted, heuristics may be used until more precise data become available.

Fourth, decision support should not be viewed as a standalone solution for improving sustainability in healthcare. Rather, it must be embedded in a broader framework (Recommendation 6) that includes pre-professional, professional, and continuing education, supportive local, regional, national, and international policies, and other systemic factors that can facilitate healthcare's journey to sustainability. CDS tools designed to guide clinicians toward more sustainable choices will be more effective if users already possess foundational knowledge about healthcare's environmental effect and their role in mitigating it. A 2020 survey of 84 international health professions institutions found that 63% offered climate-health education [ 138 ] and a 2022 survey by the Association of American Medical Colleges (AAMC) revealed that 55% of participating U.S. medical schools included the health effects of climate change in their required coursework [ 139 ]. Building on this growing educational foundation, we can reinforce climate-health principles by incorporating brief, targeted educational content directly into decision support tools [ 140 ]. In this way, clinicians will receive real-time, context-specific insights to make environmentally informed decisions at the point of care without adding to clinician burden.

AI could play an important role in building a “culture of sustainability.” While AI is often criticized for its vast energy consumption [ 54 55 56 ], it can also play a significant role in improving decision-making in the context of sustainability, e.g., by optimizing the use of resources, reducing waste, promoting energy efficiency, improving patient care with fewer resources, and reducing diagnostic errors and unnecessary testing [ 54 56 141 142 ]. As humans increasingly allow technology to guide their behavior, AI-driven decision support tools can embed sustainable practices directly into daily workflows, making environmentally responsible choices more intuitive and seamlessly integrated into standard healthcare operations.

Lastly, informatics should contribute to building local, regional, national, and global communities for advancing sustainability in healthcare (Recommendation 7). Pollution and climate change are global phenomena, and they need to be addressed by energetic, sustained, and worldwide action.

## 5. Limitations

The literature search employed in this study followed an iterative format rather than that of a structured systematic literature review. The initial search terms were derived from a previous study focused on climate change and informatics and then modified to retrieve papers addressing decision support (see *Appendix 1* ). For multiple reasons, such as inconsistent indexing, we may not have retrieved all relevant publications. Additionally, we aligned our literature review with the categories for healthcare decarbonization outlined by AHRQ [ 30 ], so not all themes relevant to healthcare sustainability, such as waste, were discussed in their own sections.

## 6. Conclusion

Healthcare's primary operating principle is “first do no harm.” The healthcare sector can improve its mission to make and keep people healthy by reducing its own pollution and carbon emissions. All levels of healthcare organizations make decisions with environmental sustainability implications, ranging from which medications are prescribed, to how facilities are heated and cooled. Yet, decision support for healthcare sustainability has largely been implemented within narrow clinical domains, such as anesthesiology and radiology. There are significant opportunities to leverage decision support for sustainability broadly across healthcare organizations–including areas beyond the scope of this paper, such as adaptation and care for patients affected by climate- and/or pollution-related phenomena. Therefore, the informatics community worldwide should craft a comprehensive and systematic agenda for developing, implementing, and evaluating decision support tools and algorithms for sustainability.

## Figures and Tables

**Table:** 

Basic search
Search: (climate change) AND (informatics) climate change: “climate change”[MeSH Terms] OR (“climate”[All Fields] AND “change”[All Fields]) OR “climate change”[All Fields] informatics: “informatics”[MeSH Terms] OR “informatics”[All Fields] OR “informatic”[All Fields] OR “informatization”[All Fields]

**Table:** 

Enhanced search
(“hospital”[All Fields] OR “clinic”[All Fields] OR “healthcare”[All Fields] OR “electronic health record”[All Fields] OR “electronic health records”[ All Fields] OR “telemedicine”[ All Fields] OR “radiology”[All Fields] OR “inhaler”[All Fields] OR “anesthesiology”[All Fields] OR “surgery”[All Fields]) AND (“ozone layer”[All Fields] OR “GHG”[All Fields] OR “climate change”[All Fields] OR “climate”[All Fields] OR “climate crisis”[All Fields] OR “carbon footprint”[All Fields] OR “greenhouse gas”[All Fields] OR “carbon dioxide”[All Fields] OR “environmental footprint”[All Fields] OR “environment”[All Fields] OR “pollution”[All Fields] OR “climate-resilient”[All Fields] OR “environmental impacts”[All Fields] OR “anesthetics”[All Fields]) AND (“decision support”[All Fields] OR “clinical decision support”[All Fields] OR “single-use medical devices”[All Fields] OR “energy efficiency”[All Fields] OR “sustainable”[All Fields] OR “sustainability”[All Fields] OR “AI”[All Fields] OR “medical informatics”[All Fields] OR “clinical informatics”[All Fields] OR “digital health”[All Fields])

## Notes

Appendix: Search strategy for papers focused on healthcare, decision support and sustainability
